# Data on metal levels in the inlet and outlet wastewater treatment plant of hospitals in Bushehr province, Iran

**DOI:** 10.1016/j.dib.2016.11.054

**Published:** 2016-11-23

**Authors:** Fereshte Babaahmadi, Sina Dobaradaran, Abdolrahim Pazira, Seyed Sajjad Eghbali, Maryam Khorsand, Mozhgan Keshtkar

**Affiliations:** a Department of Environmental Engineering Bushehr branch, Islamic Azad University, Bushehr, Iran; b The Persian Gulf Marine Biotechnology Research Center, Bushehr University of Medical Sciences, Bushehr, Iran; c Department of Environmental Health Engineering, Faculty of Health, Bushehr University of Medical Sciences, Bushehr, Iran; d Systems Environmental Health, Oil, Gas and Energy Research Center, Bushehr University of Medical Sciences, Bushehr, Iran; e Department of Natural Resources Bushehr branch, Islamic Azad University, Bushehr, Iran; f Department of Pathology, School of Medicine, Bushehr University of Medical Sciences, Bushehr, Iran; g Young Researchers and Elite Club, Bushehr Branch, Islamic Azad University, Bushehr, Iran

**Keywords:** Bushehr, Hospital, Inlet wastewater, Metals, Outlet wastewater

## Abstract

In this paper, we measured the levels of metals including Pb, Cr, Cd, Ni, Hg, Fe, and Cu in the inlet and outlet wastewater of hospitals. The samples were taken from wastewater in Bushehr׳s province hospitals, Iran. After the collection of samples, the concentration levels of metals were determined by using graphite furnace absorption spectrometer (AAS) method (Varian, SpectrAA 240, Australia). Statistical analysis of the data was carried out using Special Package for Social Sciences (SPSS 16).

**Specifications Table**

**Table:** 

*Subject area*	*Environment*
*More specific subject area*	*Metals*
*Type of data*	*Table, figure*
*How data was acquired*	*Graphite furnace absorption spectrometer (AAS) method (Varian, AA 240*, Australia*)*
*Data format*	*Raw, analyzed*
*Experimental factors*	*All wastewater samples in polyethylene bottles after acidification were stored in a dark place at* 4 °C *temperature until the metals analysis.*
*Experimental features*	*Determine the concentration levels of metals including Pb, Cr, Cd, Ni, Hg, Fe, and Cu.*
*Data source location*	*Bushehr province, Iran*
*Data accessibility*	*Data is available within this article*.

**Value of the data**

• The data presented here will be useful for the hospital managers for proper treatment and disposal of produced wastewaters in hospital.
• The data shown here may be used for health risk assessment related to hospital wastewater properties.
• Data shown here may serve as benchmarks for other groups working or studying in the field of effluent disposal, pollution control, aquatic ecosystem, and toxicology.

## 1 Data

The mean±SD concentration levels of metals including Pb, Cr, Cd, Ni, Hg, Fe, and Cu in wastewater samples in all hospital inlet samples were 0.53±0.08, 0.9±0.2, 0.035±0.008, 0.86±0.09, 0.002±0.00, 1.31±0.51, and 0.43±0.1 µg l^−1^ respectively. In the case of outlet these values were 0.5±0.04, 0.77±0.23, 0.03±0.007, 0.72±0.08, 0.001±0.00, 1.12±0.52, and 0.37±0.06 µg l^−1^ respectively. As shown in Table 1, total mean concentration levels of metals are always higher in the inlet than in the outlet wastewater. In Table 2, the value removal efficiencies of Pb, Cr, Cd, Ni, Hg, Fe, and Cu in all hospitals shown.

## 2 Experimental design, materials and methods

### 2.1 Study area description

**Fig. 1:**
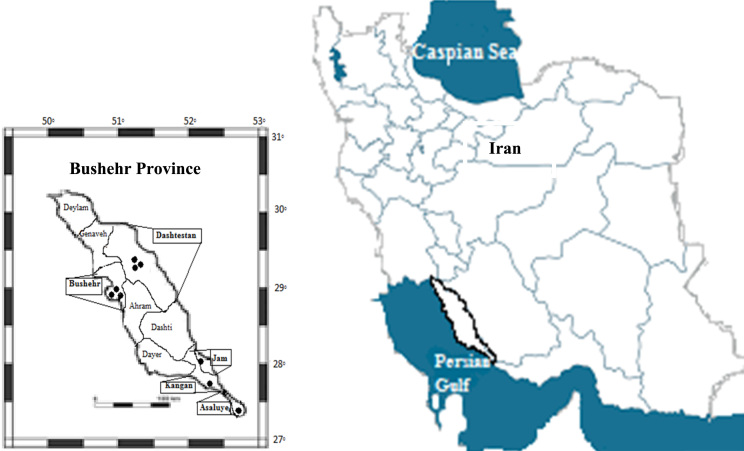
Location of selected cities in Bushehr province for sampling.

Nine hospitals in Bushehr province, Iran were selected as sampling points including Shohadaye Khalije Fars (in Bushehr), Salman Farsi (in Bushehr), Ghalb (in Bushehr), Shahid Ganji (in Borazjan), Mehr (in Borazjan), 17 Shahrivar (in Borazjan), Emam Khomeini (in Kangan), Nabi Akram (in Asaluye) and Tohid (in Jam) (see Fig. 1). In Table 3, type of wastewater treatment in every hospital is shown.

### 2.2 Sample collection and analytical procedures

In each hospital, samples were collected from inlet and outlet wastewaters monthly (in total 3 samples from every hospital). In each hospital inlet and outlet wastewaters were taken during the same days by a grab sampling method. Wastewater samples were collected by using 200 mL polyethylene bottles that were washed three times with deionized water; prior to collecting each sample, and then bottles were labeled with the sample number and location for identification. After transferring to the laboratory, all samples were acidified and stored in a dark place at 4 °C temperature until analysis. The samples were filtered by 0.45 μm Millipore filters. After that, Hg was measured by using cold vapor, and Pb, Cr, Cd, Ni, Fe, and Cu were measured by graphite furnace absorption spectrometer (AAS) method (Varian, AA 240, Australia) [1], [2].

## Figures and Tables

**Table 1:** Contents of Pb, Cr, Cd, Ni, Hg, Fe, and Cu (μg l^−1^) in wastewater samples of hospital (maximum values are expressed as bold italics; minimum values as bold underlined).

Hospital	Pb		Cr		Cd		Ni		Hg		Fe		Cu	
	**Inlet**	**Outlet**	**Inlet**	**Outlet**	**Inlet**	**Outlet**	**Inlet**	**Outlet**	**Inlet**	**Outlet**	**Inlet**	**Outlet**	**Inlet**	**Outlet**
Shohadaye Khalije Fars	0.59	0.42	1.01	0.53	0.04	0.03	0.95	***0.64***	0.003	**0.001**	***2.01***	1.04	0.48	0.32
Salman Farsi	0.51	0.40	1.02	***0.98***	0.03	**0.02**	0.93	0.57	0.002	**0.001**	1.86	***1.06***	0.43	0.31
Ghalb	**0.41**	–	0.69	–	**0.02**	–	**0.69**	–	**0.001**	–	1.98	–	**0.25**	–
Shahid Ganji	0.51	0.49	0.89	0.46	0.04	**0.02**	0.87	0.62	0.003	***0.002***	1.48	0.86	0.45	0.29
Mehr	0.46	**0.39**	**0.53**	**0.25**	0.03	**0.02**	0.79	0.48	0.002	**0.001**	0.92	0.43	0.26	**0.17**
17 Shahrivar	0.57	0.49	1.05	0.74	0.03	**0.02**	0.93	0.44	0.003	**0.001**	0.78	0.39	0.44	0.36
Emam Khomeini	***0.69***	***0.53***	***1.24***	0.83	***0.05***	***0.04***	***0.98***	0.61	***0.004***	***0.002***	1.32	1.10	0.51	**0.38**
Nabi Akram	0.58	0.47	0.97	0.48	0.04	**0.02**	0.89	**0.41**	0.003	**0.001**	**0.65**	**0.21**	***0.58***	0.34
Tohid	0.46	–	0.76	–	0.03	–	0.77	–	**0.001**	–	0.84	–	0.49	–
Mean ± SD	**0.53 ± 0.08**	**0.5 ± 0.04**	**0.9 ± 0.20**	**0.77 ± 0.23**	**0.035 ±0.008**	**0.03 ±0.007**	**0.86 ± 0.09**	**0.72 ± 0.08**	**0.002 ± 0.00**	**0.001 ± 0.00**	**1.31 ± 0.51**	**1.07 ± 0.34**	**0.43 ± 0.10**	**0.37 ± 0.06**

**Table 2:** Removal efficiencies of Pb, Cr, Cd, Ni, Hg, Fe, and Cu in all hospital (%).

Haspital	Shohadaye Khalije Fars	Salman Farsi	Ghalb	Shahid Ganji	Mehr	17 Shahrivar	Emam Khomeini	Nabi Akram	Tohid
Pb	28.81	21.56	–	3.92	15.21	14.03	23.18	18.96	–
Cr	47.52	3.92	–	48.31	52.83	29.52	33.06	50.51	–
Cd	20	37.5	–	50	37.5	37.5	20	50	–
Ni	32.63	38.7	–	28.73	39.24	52.68	37.75	53.93	–
Hg	66.66	50	–	33.33	50	66.66	50	66.66	–
Fe	48.25	43.01	–	41.49	53.26	50	16.66	67.69	–
Cu	28.81	21.56	–	3.92	15.21	14.03	23.18	18.96	–

**Table 3:** Type of wastewater treatment in every hospital.

Hospital	Shohadaye Khalije Fars	Salman Farsi	Ghalb	Shahid Ganji	Mehr	17 Shahrivar	Emam Khomeini	Nabi Akram	Tohid
Type of treatment	Activated sludg	Activated sludg	Septic tank	Activated sludg	Activated sludg	Septic tank	Activated sludg	Activated sludg	Without treatment
